# Understanding the reparative effects of schema modes: an in-depth analysis of the healthy adult mode

**DOI:** 10.3389/fpsyt.2023.1204177

**Published:** 2023-10-24

**Authors:** Duygu Yakın, Arnoud Arntz

**Affiliations:** Department of Clinical Psychology, University of Amsterdam, Amsterdam, Netherlands

**Keywords:** schema therapy, schema modes, healthy adult mode, deductive thematic analysis, qualitative approach

## Abstract

**Objectives:**

Evidence in favor of schema therapy's effectiveness in treating personality disorders is growing. One of the central and recently popular concepts of schema therapy is schema modes [i.e., temporary emotional–cognitive–behavioral states resulting from the activation of early maladaptive schemas (EMSs)]. A key aspect herein is self-reparenting, i.e., a healing relationship between the healthy adult (HA, i.e., compassionate and healthy emotional states, and functional dealing with reality) and the child modes (i.e., representation of fragile and hurt feelings and dysfunctional coping). Through an in-depth qualitative analysis, we aimed to better understand the components of the HA that enable self-reparenting.

**Method:**

Purposive sampling procedures were used to recruit eligible participants (*n* = 10) with relatively strong HA modes, as determined by high scores on positive affect and satisfaction with life measures and low scores on EMSs and psychopathological symptom measures. Semi-structured 45- to 60-min face-to-face interviews were conducted individually, in which individuals were asked to help the child modes reflected in the pictures. Interviews were then analyzed using deductive thematic analysis in MaxQDA.

**Results and discussion:**

The analysis revealed three superordinate themes comprising of a total of 10 group themes: (1) bonding between HA and the vulnerable child modes (“Bond”); (2) balancing expression and inhibition of adult and child mode emotions (“Balancing”); and (3) opposing demanding and critical voices and maladaptive coping styles (“Battle”). Furthermore, a strong HA mode seems to have a reciprocal relationship with the child modes: the HA gives nurturance and protection to the child modes, and the child modes boost up the HA with spontaneity and happiness. In conclusion, emotional stability and resilience may be highest when HA-child mode interaction happens bidirectionally; when the child modes get reparented by the HA, and the HA is informed by the child modes.

## 1. Introduction

Schema therapy (ST) was originally developed as an integrative approach to treat complex, recurrent psychological problems and is (cost-)effective for personality disorders (PDs) in both individuals (1–5) and group settings (2, 6–10). ST has been proposed and proven effective for both mood and anxiety disorders (11–14). Contemporary ST research and practice have been enriched by the recent inclusion of positive psychological factors (15–18). Concepts such as mindfulness (19), resilience (15), and compassion (20) have been proposed to be integrated into ST. However, despite the popularity of such emotional and cognitive concepts in clinical practice, they remain poorly understood within the conceptual framework of ST. An actual understanding, rather than a description, of positive factors as components of the ST framework (i.e., the HA) is crucial for understanding the effective mechanisms of change in ST and, thus, maximizing therapy effectiveness.

The fundamental aim of ST is to reduce EMSs and dysfunctional schemas that originated in early developmental stages. Various EMSs have been distinguished, and they can be defined as pervasive maladaptive patterns that underlie information processing. EMSs develop as reactions to unmet childhood needs and encompass bodily sensations, emotions, memories, and cognitions (21, 22). EMSs perpetuate themselves through dysfunctional ways of coping with them. The manifestation of EMSs within an individual may vary dramatically based on specific coping styles. Eighteen EMSs combined with three dysfunctional coping styles [i.e., surrender, overcompensation, and avoidance; (21); later relabelled as resignation, inversion, and avoidance; (6)] can create very different momentary reactions, described as schema modes. These momentary reactions help the individual deal with schema-related emotions in the short term, but they eventually lead to needs not being met and schema confirmation. By contrast, the theory states that functional coping with schema activation can help with getting needs met and schema disconfirmation. Functional coping with schema activation is attributed to the healthy adult mode, which is usually weak in severe psychopathology.

ST practice and research has increasingly focused on the different states the individual can experience as a result of specific ways of coping with EMSs activation. That is, schema modes (SMs) were introduced to conceptualize the complex combination of the patient's prevalent emotional states, schemas, and coping responses to work with them more efficiently (23–25). An SM formulation of PDs provides a well-defined model that (1) elucidates the current state of the individual, (2) enables a precise formulation of sudden changes in emotional state and behavior, (3) reflects the connection between schema modes and behavioral manifestations of psychopathological symptoms, and (4) allows for therapy to be tailored to the characteristics and needs of each patient. As such, the SM model may aid in the recognition and validation of the patient and their problems, allowing the therapist and patient to focus on concrete therapeutic goals and bring about symptom severity reduction (26).

Modern ST primarily focuses on SMs, has specific techniques for addressing different modes, and aims to ultimately “heal” the child modes (the SM that results from resignation to painful EMSs) and strengthen the healthy adult mode (the SM that represents the healthy part of the patient). During ST, the therapist built up a secure attachment with the patient in which (s)he could help the patient meet the unmet early childhood needs while staying within the professional relationship boundaries (i.e., limited reparenting). Thus, the therapist supports and encourages HA to take care of the needs of the child mode.

ST ultimately aims to help patients strengthen their HA mode, recognize their core emotional needs, and heal EMS underlying child modes. Gradual internalization and integration of reparative parenting provided to the child modes by the therapist in the patient's own HA have, as such, been recognized as an effective mechanism of ST. Ideally, at the end of therapy, the HA mode functions as a “good enough” parent to fulfill a variety of unmet needs. Therefore, ST helps patients develop their HA to identify a problem, employ a different perspective, and then select and apply a healthier strategy to meet unmet child needs. Thus, the function of the HA is argued to be central to change in schema therapy (16, 27, 28). Similarly, the importance and centrality of the vulnerable child (VC) mode were established both theoretically (21, 22) and empirically (28–30). The VC contains emotionally painful experiences and distressing emotions, and it can be very painful to stay in contact with the VC. Next to the internalizing responses characterizing the VC, externalizing dysfunctional responses can occur, which are represented by other child modes (i.e., angry, impulsive, and undisciplined child modes). HA needs to be able to deliver good parenting to all the child modes to establish symptom recovery. Hence, it is important to understand the role of the HA in mental health and wellbeing. Although it is established that an effective treatment, i.e., decreasing PD severity, is established mostly through the HA and the VC [i.e., HA plays a role in recovery, both directly and through reducing the VC strength, and a reduction in VC strength leads to an increase in HA; (28)], we do not have a detailed understanding of how these modes influence each other and the roles of the other child modes in this. Relatedly, Louis et al. (17) argued that positive schemas (i.e., schemas of HA) are not the mere opposite of negative schemas (i.e., schemas of VC). Similarly, the HA and the child modes represent healthy and pathological aspects of the individual. They thus need to be evaluated as separate dimensions, each contributing to changes during treatment. This implies that *connections* between SMs may, at the very least, be as important as the SMs themselves. Little is known about these connections. In a recent study, the network of schema modes was studied, and although VC was central in the network, HA was not central. However, although the HA was not central in the network of maladaptive modes, it was the most important schema mode to differentiate people with personality disorders from healthy participants (30). This is in line with Louis et al.'s (17) claim that positive schemas are not necessarily mere opposites of maladaptive schemas. Moreover, despite HA and VC not being each other's opposites and not being strongly related in a transversal network of schema modes, they influence each other during the change process of treatment (28). This raises the question of how these influences take place.

However, to understand the dynamics of the HA–VC connection, the other child modes need to be considered. This is because primary responses might be more in the externalizing, acting-out domain, than in the internalizing domain, in which the VC is classified (6). Thus, EMS activation might lead to patients shifting into externalizing child modes, such as the angry child, the enraged child, the impulsive child, and the undisciplined child modes (6, 31, 32). According to the ST model, the HA also has an important task to set limits on uncontrolled acting out of these modes and to express compassion to the self for the need for frustration that is connected to their activation. In addition, the HA is supposed to acquire the capacity during treatment to liberate the person from dysfunctional high standards and (self-)punitiveness (i.e., the Demanding and Punitive Parent modes). Similarly, the HA has a task in correcting the dysfunctional use of so-called “Coping Modes”, in which the person's feeling, thinking, and behaving are dominated by attempts to avoid schema activation (avoidance) or to fight the schema by pretending its opposite (inversion). In sum, the HA should become responsible for regulating the frequency, intensity, and duration of the maladaptive schema mode switches and encourage the individual to find and keep the balance within themselves. The HA is thought to achieve such by (1) nurturing, affirming, and protecting the VC, (2) using self-discipline and setting limits to the angry/enraged child and impulsive/undisciplined child modes, and (3) limiting maladaptive coping modes and dysfunctional parent modes [(22), p. 278]. Although such “tasks” of the HA are well-defined within ST (33), there is still a lack of understanding regarding how the HA mode operates in relation to the child modes to induce effective change.

In summary, the HA mode and its relation to VC are critical elements in ST. However, there is a lack of detailed insight into what exactly the essential elements of the HA relevant for functionally dealing with the child modes are. We, therefore, aimed to better understand the HA mode when dealing with the activation of the child modes. We started with a detailed conceptualization of its construct based on empirical evidence. Furthermore, we aimed to investigate the overlap between the theoretical description of the HA and the idiographic representations of the construct in relation to the child modes as elicited from descriptions by highly functional laypeople. To gain a thorough knowledge, we used a qualitative and explorative approach. As we started from the theoretical framework of ST, our interpretation of the participants' ideographic experiences was not merely data-driven; our approach was both data-driven and theory-driven. Thus, we opted for a mixed approach, using qualitative thematic analysis (34, 35).

## 2. Materials and methods

### 2.1. Preliminary study

#### 2.1.1. Participants

Data were collected from 88 (40.6%) male participants and 129 (59.4%) female participants between the ages of 19 and 58 (M = 30.72, SD = 8.52). Most of the participants (72.8%) reported medium or high income. A total of 82 (37.8%) participants were single, 52 (23,9%) participants were in a relationship, 67 (30.9%) participants were married, and 15 (6.9%) were divorced or widowed at the time of data collection. The majority of the participants (75.6%) had never psychological help before in their lives.

#### 2.1.2. Instruments

##### 2.1.2.1. The Young Schema Questionnaire—Short Form 3 (YSQ-S3)

YSQ-S3 (36) is a 90-item questionnaire that assesses 18 EMSs that can be grouped into five domains (21). The YSQ-S3 has acceptable levels of overall reliability and validity for Turkish adolescents and adults specifically (37, 38). In the present study, the alpha coefficients of the schema domains were found to range from α = 0.72 (overvigilance/inhibition) to α = 0.88 (disconnection/rejection), with a total scale alpha coefficient of α = 0.94.

##### 2.1.2.2. The Brief Symptom Inventory (BSI)

BSI (39, 40) is the shortened version of the symptom checklist (SCL-90-r) assessing psychopathological symptoms during the past 7 days based on an individual's response to 53 items (e.g., feeling fearful and mind going blank) using a 5-point likert scale (0 = not at all and 4 = extremely). The Turkish translation used in the current study previously revealed acceptable levels of reliability and validity (41). In the present study, the internal consistency of the whole scale was α = 0.95, with subscale consistencies ranging from α = 0.78 (hostility) to α = 0.88 (depression).

##### 2.1.2.3. The Satisfaction with Life Scale (SWLS)

SWLS (42) is a five-item self-report instrument using a 7-point likert scale (1 = strongly disagree and 7 = strongly agree). The scale yields a total score, for which scores between 0 and 20 indicate the level of dissatisfaction (i.e., a lower score indicates more dissatisfaction with life), whereas scores above 20 indicate the level of satisfaction (i.e., a higher score indicates more satisfaction with life). Acceptable reliability and validity were reported for the Turkish translation of the SWLS (43). In the present study, the internal consistency of the scale was found to be α = 0.84.

##### 2.1.2.4. The Positive and Negative Affect Schedule (PANAS)

PANAS (44) is a 20-item self-report instrument using a 5-point likert scale (1 = very slightly or not at all and 5 = extremely). Acceptable reliability and validity were reported for the Turkish translation of the PANAS (45). In the present study, the internal consistency of the scale was found to be α = 0.82 for the NA and α = 0.87 for the PA.

#### 2.1.3. Procedure

We used the snowball sampling procedure to recruit a diverse group in Istanbul, Türkiye. The first author gave course credits to the students to encourage people to fill out the questionnaires via online platforms (i.e., Facebook, Instagram, and e-mail). All participants filled out an online survey after consenting to participate. Inclusion criteria were being within the age range of 18–70, living in Istanbul (or being able to come to have the interviews in person in Istanbul), and having the ability to understand, read, write, and speak Turkish. The exclusion criterion was not being able or willing to sign an informed consent. No compensation was given for participation. The study and methodology were approved by the Applied Ethics Research Center of Istanbul Arel University in Türkiye.

### 2.2. Qualitative study

#### 2.2.1. Participants

Participants were recruited through a purposive sampling procedure to increase the prevalence of the themes associated with the HA mode (46). We aimed to reach people with a relatively healthy mental state; therefore, we calculated sum scores and identified participants who scored low on dysfunctional questionnaires (i.e., EMSs, negative affect, and psychopathological symptoms) and high on functional questionnaires (i.e., high scores on positive affect and satisfaction with life). We first selected participants who were both in the top 40% of functional scores and in the lower 40% of dysfunctional scores. We wanted to avoid selecting people who might provide socially desired answers or deny any problems. Therefore, we excluded people who scored in the highest 5% of functional and/or lowest 5% of dysfunctional questionnaires. All 39 selected participants were individually called by the first author with respect to their scores to invite them to the study. Fifteen participants were successfully reached by phone and were available for an interview. A total of 10 participants agreed to participate. Interviews were carried out with five men and five women between the ages of 22 and 40 (M = 32.40, SD = 7.70). Their income ranged from medium (%30) to high (%70). Eight participants had never had any psychological help before in their lives, and two participants received individual psychotherapy for a short time. Five people were in a relationship at the time of the interviews, four people were single, and one participant did not want to answer this question.

#### 2.2.2. Procedure

The present study's procedure comprised in-depth, semi-structured interviews. Following the advice of Lobbestael et al. (26), who suggest using experiential techniques to trigger schema modes, the participants were exposed to images that portray children who are in need of parenting, with the instruction to tell how they would respond to the child.

Eight cards depicting a child (i.e., lonely child, abandoned child, abused child, humiliated child, angry child, impulsive child, undisciplined child, and happy child) from the iMode Cards (47) were utilized to trigger how the healthy adult mode of the participants would react to the portrayed child modes. Moreover, a card depicting the HA mode was also used as a projective tool for the participants to describe their understanding of a healthy adult. The interview started with questions related to how participants deal with stress evoked by the triggering of the EMSs in general (see Appendix A for all the interview questions). Participants were encouraged to express their ideas freely. Next, the HA card was presented, and the participants were asked to describe the person portrayed in the picture. Later, the participants were presented with the child cards and asked to help the children pictured on the cards. Participants were encouraged to identify the children's needs and emotions and describe how they would support them. The happy child card was presented last to find out what participants' understanding of a healthy child was. Interviews lasted between 45 and 60 min. All participants in the sample that participated in the qualitative study were interviewed individually by the first author at the Istanbul Arel University Clinical Psychology Unit. All interviews were audiotaped and transcribed. Subsequently, the data were analyzed in line with the rules of thematic analysis. We used MAXQDA 2022 (48) for data analysis. The study and methodology were approved by the Applied Ethics Research Center of Istanbul Arel University in Türkiye because, at the time of the data collection, the first author was also affiliated with Istanbul Arel University, and the data were collected in Türkiye.

#### 2.2.3. Data analysis

The data analysis procedure was conducted according to Braun and Clarke's (34) guidelines for thematic analysis (see Table 1). Following Kuckartz (35), the coding process was conducted sequentially, working on the text section by section. Kuckartz (35) suggests that at least two coders should be involved in the coding process. Thus, a research intern qualified in clinical psychology also coded the main themes concurrently. Following King (49), we started with the predefined codes that are related to the definition of the HA. When the coders agreed on the final definition of the themes, a third coder who also specialized in clinical psychology coded the interviews one final time using the code book, and the interrater agreement was calculated on these scores.

**Table 1:** Process of thematic analysis based on the six-phase guide of Braun and Clarke (34).

**1. Data familiarization**
Transcriptions were done by the first author. The entire dataset was repeatedly read by the first author to identify recurring meanings and patterns and familiarize with the data. Definitions of categories reflecting adaptive responses to the child depicted on the cards were refined, and memos were created for particularly interesting topics.
**2. Generating codes**
The reactions of the participants were categorized via theoretical interpretation. The coding process was conducted sequentially, working on the text section-by-section. Initial list of possible codes was created based on the responses of the participants and on Young's conceptual framework of the functioning of the HA mode.
**3. Searching for themes**
Following the suggested steps, consensual coding (i.e., two coders code the same text separately and then discuss their results together to reach a consensus in category definitions and coding) was done. During consensual coding, weekly meetings were held in which the coders shared their thoughts and ideas and broadened their understanding of the data. The changes regarding the codes were written down and the coding books were merged.
**4. Reviewing of themes**
Finally, two coders came together and reviewed the entire dataset, as well as the coded data extracts. The themes were developed by the first author. Then the final themes were discussed with the second author to double-check the internal validity of the classification. Themes were defined by reviewing their contents in detail. Various changes were made to establish an agreement between the coders and the second author.
**5. Definition of the themes**
The broader themes and the subthemes were identified and coded by the two coders. A third coder also coded the whole interviews only based on the code book. Interrater reliability scores were calculated.
**6. Writing of the article**
The context and the structure of the newly generated codes and themes were finalized. Literature search was done to discuss the newly developed items in relation to the relevant existing empirical and theoretical work.

#### 2.2.4. Trustworthiness of the study

The internal validity of the themes is one of the most crucial points in thematic analysis (35). Efforts were made to ensure the trustworthiness of the study. First, we created a code manual stating the initial coding, which later became the codebook, to increase the credibility of the study (50). Moreover, consensual coding with another researcher and debriefing with the second author were used to further increase credibility (51). After the identification of the themes by the separate coders, interrater agreement was calculated using Cohen's kappa with the text coded by a third rater.

## 3. Results

The analysis produced three main themes, and each theme had two subordinate themes (see Table 2 for a list of themes and subthemes with Cohen's kappa coefficients).

**Table 2:** Themes, agreement rates, and Cohen's Kappa coefficient.

**Themes**	**Agreements**	**Disagreements**	**Total**	**Percent**	**Kappa**
Bond	162	32	194	83.51	0.78
Compassion and nurturance	108	35	143	75.52	0.67
Emotional attunement and encouragement	36	16	52	69.23	0.59
Balance	164	15	179	91.62	0.89
Setting boundaries and providing space	122	10	132	92.42	0.90
Self-reflection and expressing emotions	40	7	47	85.11	0.80
Battle	104	17	121	85.95	0.81
Hope and faith	38	8	46	82.61	0.77
Embracing challenges and self-empowerment	62	13	75	82.67	0.77
Total	836	153	989	84.53	0.83

### 3.1. Bond: the HA's ability to take care of the needs of the VC

The most common approach among participants was trying to get in touch with the children represented in the iModes. Thus, the first dimension of a healthy adult involves kindness, understanding, and warmth toward lonely and abandoned children and emotional attunement to the children's emotions.

#### 3.1.1. Compassion and nurturance

Participants reacted to the abandoned and lonely children portrayed in the images with connection, compassion, and nurturing.

“*She needs to understand herself and find her own way of living. But this takes time, and she needs more time. First, she needs to be compassionate toward herself. She should be able to calm herself down*.” (Interviewee 1)

Most of the participants also took it a step further and stressed the importance of providing care via physical connection:

“*You should talk to him, try to love him, try to give confidence to him not via just talking, but also touching and feeling. I think this is the most effective thing. You can just say it from a distance, but it might not be effective but if you hug him and reinforce this feeling, then the person might feel stronger”* (Interviewee 2)

#### 3.1.2. Emotional attunement and encouragement

Some participants employed a stronger emotion-focused approach to support the troubled children portrayed in the images. One participant pointed out the importance of not just channelizing the child in a certain direction that is good for the child but being more attuned to the emotions of the child and being guided by them.

“*I would sit next to her, attuned to her. It's like more than what I want to do or say, what she would like to say, to encourage the things inside her to come out. For her to talk about her emotions and do what she would like to do. Just to make her feel that I am with her. Anything can happen but I will still be with her. To give her the confidence that you can try or do anything you wish, and if there is a problem, you will find me here… Thus, it is not possible to just pull her from her hand… First somebody needs to be with her, attuned to her and then do something when her emotions are neutralized… For example, instead of a parent standing and talking, you should sit next to her. If she sits on the ground, you should also sit next to her and feel the cold that comes from the ground. Try to understand her, feeling and trying to understand…”* (Interviewee 4)

This participant seems to point out the difference between connecting with the child portrayed on the cards with genuine trust and respect and mostly relying on herself as a source of information. In that sense, the participant seems to point out that she needs to understand, with the help of the child, where it hurts and decide, with the guidance of the child, what is the right thing to do.

### 3.2. Balance: the HA's ability to set limits to AC and IC and provide space for the needs of the VC

When dealing with the angry and impulsive children portrayed on the cards, almost all the participants mentioned the importance of setting limits from a caring perspective without being punitive. Thus, the second dimension involves healthy expressions and reactions to deal with the impulsive and angry children represented in the images. As such, it aims to decrease self-destructive reactions and find a balance while fighting with negative emotions.

#### 3.2.1. Setting boundaries and providing space

Almost all participants highlighted the need for setting limits for the children pictured on the cards so that they can stop them from hurting themselves. Most behaviors to set limits seem to happen at the cognitive level, with participants taking charge to stop the child from doing what (s)he was doing and distracting the child from doing it (i.e., to release negative energy through sports, hobbies, and social gatherings).

“*Parents should build appropriate limits. He shouldn't get everything he wants because if he doesn't understand the limits, he cannot decide how to cope with a situation in which he wants something but cannot get it. This is not good for him in later life.”* (Interviewee 3)

#### 3.2.2. Self-reflection and expressing emotions

Another way of creating balance expressed by the participants is to increase their interoceptive awareness and encourage them to express their current emotions. In this regard, most participants mentioned emotion regulation strategies that involve tuning in to what is happening at the emotional level and creating space for their emotions:

“*Rather than distracting myself from the problem, I prefer to live through that emotion and reflect on it. That's how I deal with it… If I am too sad, I cry. Sometimes it lasts one or two days… If I feel angry, I let myself feel it*, if I am sad, I let myself feel it as well. I don't want to keep it inside. I try to live it like that…” (Interviewee 4)

While everybody seems to be mentioning the importance of expressing emotions, one participant mentioned following the negative emotion itself and allowing the emotion to come out to acquire emotion regulation.

“*Before, I was always looking for training or therapy. I wanted to share this painful space with somebody I know, and I trust that (s)he knows it better than I do. But for the last 2 years, it is more like achieving to be able to stay with myself. I realize if I turn to other people, I always look for an approval, an answer… Now I turned to the wisdom within. I don't turn to anybody to ask, there is no new interaction, thus, I let this flow out of me, discovering the answer within the insight that comes to me… If I am angry, I write things and then rip it off, or I punch the pillows and put myself on the ground, I say to myself ok, I am grounding, I sit, I put my head on the ground. I feel like I am praying… There is nothing coming from outside, only I am present at that moment…”* (Interviewee 4)

### 3.3. Battle: the HA's ability to moderate maladaptive modes to defend the VC

The third dimension of the healthy adult involves strategies aiming to battle maladaptive parents and coping modes to bounce back from negative experiences. In this theme, most participants reflected expressions and reactions that involve realizing one's own strengths and capabilities, being aware of their inner power, keeping a positive stance toward life, and dealing actively with challenges.

#### 3.3.1. Hope and faith

The theme of hope emerged distinctively across all interviews. People wanted to give hope to the troubled children that their lives would not continue like that, and they would find a way to survive and make it better. They seemed to be worried about the portrayed children starting their lives without enough support and hope for their future.

“*The definition of health might vary, but I think being healthy is to love herself and life. The most crucial part is hope… To be able to keep hoping the best for your future… I think this child shouldn't be starting something without hope at a very early age.”* (Interviewee 5)

Some participants expressed the importance of hope in their religious beliefs, while others connected it more to their belief system in general.

“*It actually works really well for me to assure myself that ‘this too shall pass' I will try to find a way out because I believe, there is always a way out. It always happened this way from the beginning. Either I found a solution, or I got help from somewhere somehow. I believe that it gives you energy to have optimism. That gets me out of troubles”* (Interviewee 6)

#### 3.3.2. Embracing challenges and self-empowerment

Participants often emphasized the importance of persisting in the face of challenges with a constructive and realistic evaluation of the problems. Some participants also focused on relying on their own strength and decisions when they are faced with challenges, although they felt that they were not getting enough support.

“*Life is good for me, challenging, but you must keep swimming as the waves grow high… My life has been always challenging but I still am able to do everything that I wanted to do… because I used everything I came across as a lesson…”* (Interviewee 5).

Some people expressed that they embrace the challenges and believe that they have enough power to deal with them, although there might not be so many people on their side.

“*People with what is called ‘intrepidity' are a little more combative (in the face of hardship). People who have lots of hardship in their life tend to be not so catastrophic, they focus on how to get out of it (the problematic situation), or what they can do about it”* (Interviewee 5).

### 3.4. Insights for the process of eliciting the subthemes

In this study, we aimed to look for themes related to the three dimensions of healthy (re)parenting proposed by Young et al. (22), in the early theorization of schema theory. However, because of the nature of the qualitative analysis, reading the text so many times and updating the theme structure repeatedly leads us to different questions and formulations to understand how the HA functions. We came to the understanding that the first group of themes (i.e., compassion and protection, setting boundaries and providing space, and hope and belief) involves the child relying on another strong person who has the knowledge of what to do when a child is in need. In other words, the flow of care is top-down. We found in our previous study that the relationship between the HA and the VC is reciprocal, in the sense that if a decrease is acquired in the VC, it will lead to a change in the HA as well, and vice versa (28). Inspired by that, we realized after the creation of the main code system that the HA–VC relation is reciprocal (i.e., not only top-down, in which the HA just *helps* the child feel better, but the HA also *changes* with this interaction). This might suggest that there is also a bottom-up effect, and the HA might be influenced by the spontaneity and creativity of the child. The spontaneity and creativity of the child can be transferred to a source of energy and intuition in adult life.

We concluded that the second group of subthemes (i.e., emphatic attunement, self-reflection and expressing emotions, and embracing challenges and self-empowerment) are not necessarily *only* related to comforting the child but also imply a direct focus on emotions and using them as a source of direction. This seems to be more of a bottom-up process as it utilizes the information (i.e., the core of the unmet needs) that comes from the portrayed children. This approach seems to give genuine respect and credit to the child and emphasizes togetherness in the relationship between the HA and the child modes. As togetherness increases, the HA's ability to understand, verbalize, and acknowledge the needs and the emotions resulting from the unmet needs also improves.

## 4. Discussion

The HA has a central role in the practice of schema therapy as the aim of schema therapy is to get the HA to provide good parenting to the child modes and create a healthy emotional equilibrium in the relationship between the modes (21, 22). Although the HA is accepted as one of the most important pillars of mechanisms of change in schema therapy, there is still limited insight into what contributes most to change. Previously, we empirically established the interplay between schema modes in inducing a change in personality disorder symptoms. We found that both HA and VC are important in the change process, while HA and VC reciprocally influence each other (28). Following our previous study, we aimed to have a better understanding of how the HA functions to facilitate change. We investigated the responses of people with good mental functioning toward children in different emotional states depicted on cards, assuming that these responses would be representative of how the HA mode could deal with a person's inner maladaptive child modes.

The main themes of the current study were built upon the earliest definition of the HA in schema theory (22). Accordingly, the main function of the HA is to provide nurturing and protection to the VC, setting limits for the angry child, the impulsive child, and other maladaptive modes, and fighting with the dysfunctional parent modes. The results revealed a good agreement on the presence of these three themes in the interviews. There are slightly different definitions and classifications of the HA in the literature (33, 52). However, most of the research agrees that HA should help reduce the frequency and duration of maladaptive modes, and HA growth is often rated on this basis. To formulate these functions, we identified the three B's (i.e., bond, balance, and battle) of the HA. Although we established a good agreement on these themes, reading the interviews also provided a nuanced insight that the relationship between the HA and maladaptive modes might be more complex than previously thought as we found evidence for the healthy function of exploring and becoming aware of the unmet needs underlying the maladaptive mode. This awareness informs the HA so that it further grows and can take actions necessary for mental wellbeing.

Thus, although our inquiry started with the earliest definition of the HA, we observed bottom-up processes next to the hypothesized top-down processes, and we organized subthemes based on this understanding. For instance, most of the participants replied that they approached the troubled children withholding, loving parental care, and protection. This care probably comes from their own internalized good parent who wants to provide care to the children (i.e., the flow of care goes from parent to child in a top-down way). The parent knows how (s)he needs to provide the care and what is good for the child. This way of (re)parenting is well recognized and commonly addressed in schema theory (21, 22, 33). However, other participants tried to relate to the child at a different level (i.e., the process is more reciprocal with a lot of empathic listening and tuning in with the bottom-up processing of emotions). Thus, there seems to be an important diversity in the nature of the responses as they varied widely in the study. One could argue that good parenting includes empathic reactions and tuning in by its nature and definition. However, our results implied a more far-reaching process. During the interview, participants reported that they got a lot of *support and fulfilment* for themselves from the depicted child when they connected with them. There seemed to be a feeling of togetherness and a concurrent change in the process. This was different from some participants reporting they simply liked it because they helped somebody, and they felt good about that. It is more as if they experienced personal growth by connecting with the child. To our knowledge, the difference between connecting with the child modes in top-down and bottom-up ways has not been theoretically discussed before. Thus, the HA being fulfilled by the spontaneity, creativity, and imagination of the child modes is often overlooked in the theory and perhaps also in clinical practice. Without attending to this, there might be limited room for the HA to benefit from connection with the child modes for its own growth.

### 4.1. Insights from Leary's interpersonal circumplex

The top-down and bottom-up aspects of the HA–child mode connection can be placed within Leary's (53) conceptualization of the multidimensional character of personality psychology. Leary argues that there is a two-dimensional representation of interpersonal space. The vertical axis runs from dominance to submission (i.e., a structure of control that goes from top to down), and the horizontal axis from hate to love (i.e., love implying intimacy and fellowship) [see Leary (53), p. 65 for a figure representation of the 16 generic interpersonal themes in the two-dimensional space defined by these axes]. The top-down processes of HA reparenting are more characterized by dominance (though love is certainly a part of it), whereas bottom-up processes are characterized more by reciprocity, hence less dominance. In terms of the first theme, although compassion and protection themes are very high on the love dimension, they can still be placed higher in the dominance axis compared to having empathic attunement and tuning in with the emotions. Similarly, setting boundaries relates more to dominance, while reflecting on emotions can be placed closer to the dimension of love. Finally, expecting change through hope and faith is high on dominance, in a way that the change is based more on an externalized factor, while embracing challenges and empowering self implies more togetherness and common humanity, hence closer to the love dimension.

### 4.2. Clinical implications

It has been empirically shown that although the HA is not central to the network of maladaptive modes, it is still the most important predictor to differentiate people with personality disorder problems from those without a diagnosis of PD (30). Moreover, it plays an important role in the change process during treatment (28). Thus, understanding the different aspects of the HA seems to be important to achieving better results in treatment. Schema therapists “train” the HA in therapy to develop in such a way that it can validate emotions, provide reasoning, build limits, give hope, and combat critical and demanding voices. However, the outlook for HA per individual might vary based on the frequency and intensity of other (maladaptive) modes. In this regard, working on the HA is like trying to alter an ecosystem altogether rather than working on one mode in particular. Thus, the aim is not just to develop the HA so that it can cope with the maladaptive modes, but to create *a new way of relating* between modes by working on the balance between the top-down and bottom-up processes. In this way, we can also discuss the growth of the HA based on the quality of its connection to the maladaptive schema modes. Different maladaptive modes require different approaches from the HA. For example, a HA approach high in dominance implies a risk when dealing with the VC mode by not understanding the underlying need and taking appropriate action, while it is very useful to create safety and protection from the critical and demanding internal voices.

Several limitations should be considered when interpreting our results. First, although we sampled the participants from a larger group based on their wellbeing measures, the interviews were conducted with a few Turkish participants. As with most qualitative studies, this study was explorative and did not aim to test prior hypotheses. The aim was to explore what people feel is central to a healthy adult mode. Hypotheses can be derived from such an exploration and tested in future studies. Second, cultural factors (i.e., linguistic nuances considered while rating and creating themes, the parental rearing styles that are embedded in a culture) are very important in terms of interpreting the results, and the frequency of the coded subthemes could vary very much based on the cultural background of the researcher and the participants. Thus, the generalizability of the current theme system needs further research, especially in cross-cultural samples. Third, we assumed that responses toward children depicted on cards can be generalized to healthy responses that people make toward their inner emotional states, described in schema theory as modes. It would be interesting to directly study how people high in wellbeing deal with their schema modes and the associated unmet needs.

To conclude, the present study strives to understand the structural functioning of the HA, and the main themes were identified as bond, balance, and battle. Furthermore, each main theme was divided into two subthemes. The first group of subthemes (i.e., compassion and protection, setting boundaries and providing space, and hope and belief) is high in dominance and provides top-down care for the child modes, whereas the second group of subthemes (i.e., emphatic attunement, self-reflection and expressing emotions, and embracing challenges and self-empowerment) is more reciprocal, open for bottom-up information from the child modes, and has an understanding of togetherness, which implies learning from the child and changing together. It is important to further elaborate on this two-level structure of the HA within its three main functions to better understand the mechanisms of change in schema therapy. It is also important to investigate the change in the HA during therapy in terms of top-down and bottom-up dimensions for future studies. It might be the case that different phases of therapy might benefit from a more fluid version of the HA by fine-tuning dominance and love dimensions.

## Data availability statement

The original contributions presented in the study are included in the article/Supplementary material, further inquiries can be directed to the corresponding author.

## Ethics statement

The studies involving humans were approved by Applied Ethics Research Center of Istanbul Arel University. The studies were conducted in accordance with the local legislation and institutional requirements. The participants provided their written informed consent to participate in this study. Written informed consent was obtained from the individual(s) for the publication of any potentially identifiable images or data included in this article.

## Author contributions

DY collected the data, performed the analyses, and wrote the manuscript. AA revised and corrected the manuscript. DY and AA discussed the ideas presented in this paper together. All authors contributed to the article and approved the submitted version.
