# Supplementary material for: Understanding the reparative effects of schema modes: an in-depth analysis of the healthy adult mode
Source: Front Psychiatry. 2023 Oct 24;14:1204177. doi: 10.3389/fpsyt.2023.1204177 (PMC10628052; doi:10.3389/fpsyt.2023.1204177)
Supplement: Supplementary file 1 [file Table_1.pdf]

Appendix A (Interview Questions)

Introduction questions	- How is your life going?<br>- When you face a problem in your life, how do you deal with it?<br>- When handling a problem, what are your best characteristics that help you to solve it?<br>- Let's say there is a problem, and you can't come through it. How do you deal with that?<br>- What are the most important characteristics of a person who copes well with most of the problems?
Questions for the HA Card	- Now, this is our first card. What do you think about the person on this card?<br>- Let's say this person has a problem. What kind of a problem could it be?<br>- How does this person handle this situation?<br>- Which personality traits would help this person the most in dealing with the problem?
Questions for the cards of<br>Abandoned Child<br>Angry Child<br>Lonely Child<br>Impulsive Child<br>Humiliated Child<br>Undisciplined Child<br>Abused Child	- How does this child feel?<br>- What does this child need?<br>- If you were to help this child, what would you do?<br>- Which characteristics should this child's parents have to help this child?
Questions for the Happy Child Card	- This is our last card. How do these children feel?<br>- What kind of parents do you think these children have?

Questions for the Favorite Card	- Why did you choose this card?<br>- What would be the most important characteristics of a person who can parent this child.
